# Environment and mate attractiveness in a wild insect

**DOI:** 10.1093/beheco/arac067

**Published:** 2022-07-26

**Authors:** Tom Tregenza, Petri T Niemelä, Rolando Rodríguez-Muñoz, Paul E Hopwood

**Affiliations:** Centre for Ecology and Conservation, School of Biosciences, University of Exeter, Exeter, UK; Organismal and Evolutionary Biology Research Programme, University of Helsinki, Viikinkaari, Biocenter, Helsinki, Finland; Centre for Ecology and Conservation, School of Biosciences, University of Exeter, Exeter, UK; Centre for Ecology and Conservation, School of Biosciences, University of Exeter, Exeter, UK

**Keywords:** among year, attractiveness, environment, sexual selection

## Abstract

The role of female choice in sexual selection is well established, including the recognition that females choose their mates based on multiple cues. These cues may include intrinsic aspects of a male’s phenotype as well as aspects of the environment associated with the male. The role of the spatial location of a potential mate has been well studied in territorial vertebrates. However, despite their role as laboratory models for studies of sexual selection, the potential for insects to choose their mates on the basis of location has scarcely been studied. We studied a natural population of individually tagged crickets *(Gryllus campestris)* in a meadow in Northern Spain. Adults typically move between burrows every few days, allowing us to examine how pairing success of males can be predicted by the burrow they occupy, independent of their own characteristics. We observed the entirety of ten independent breeding seasons to provide replication and to determine whether the relative importance of these factors is stable across years. We find that both male ID and the ID his burrow affect the likelihood that he is paired with a female, but the burrow has a consistently greater influence. Furthermore, the two factors interact: the relative attractiveness of an individual male depends on which burrow he occupies. Our finding demonstrates a close interaction between naturally and sexually selected traits. It also demonstrates that mate choice studies may benefit from considering not only obvious secondary sexual traits, but also more cryptic traits such as microhabitat choice.

## Teaser

We show that female insects choosing to cohabit with a male place more importance on where he lives than on who he is, but the combination of the two is more important still. We know that female birds often choose a male based on the quality of his territory; our 10 years of observing crickets moving around a Spanish meadow to share burrows with members of the opposite sex, reveals insects can do the same.

## INTRODUCTION

Females are known to choose their mates based on multiple aspects of male phenotype (Rosenthal 2017). Experimental studies of insects and fish have been crucial to establishing the central role of female choice in sexual selection. However, the small size, and often short lifespans of these laboratory model animals has meant that it has been difficult to determine whether the traits identified as targets of sexual selection in the laboratory are also the most important factors in nature. Studies of the multivariate nature of mate choice in the wild have been dominated by studies of birds and a few other vertebrates, and have identified male territory quality as an important determinant of male mating success in the wild (see Candolin [2003] for review). Among the earliest such studies was Alatalo et al’s (1986) study of pied flycatchers *(Ficedula hypoleuca),* which argued that females choose territory quality and not male characteristics. Subsequent studies have demonstrated that in flycatchers and other territorial birds, females do indeed choose males on the basis of territory, but other aspects of male phenotype also play a role (e.g., Lambrechts and Dhondt 1988; Lifjeld and Slagsvold 1988). Similarly, female Lake Victoria cichlids, *Pundamilia nyererei* choose mates on the basis of both intrinsic qualities of the male and features of his territory (Dijkstra et al. 2008), and both male phenotype and territory quality influence female choice in the puku (*Kobus vardoni)*, and topi (*Damaliscus lunatus)*; antelope species in which males defend resources but are not involved in parental care (Balmford et al. 1992). The consensus appears to be that females use intrinsic features of male phenotypes, as well as aspects of his extended phenotype (Dawkins 1982) in mate choice decisions (Candolin 2003).

These findings raise two questions: First, are intrinsic male features consistently more or less important than features of the location the male occupies? Or do they vary substantially over time (as we might expect if direct benefits of particular locations depend on factors such as population density and the weather)? Second, are there interactions between intrinsic male traits and features of the area that the male inhabits that affect how attractive the male is to females? For instance, are males that are more successful in attracting females in one area also more attractive in another area? These potential interactions between a male and his environment affecting his success in sexual selection relate to broader potential genotype by environment interactions (GxE). GxE affecting the expression of sexual traits have been demonstrated through laboratory studies in a range of species (Hunt and Hosken 2014) from bulb mites (*Rhizoglyphus robini*) (Plesnar-Bielak et al. 2018) to guppies (*Poecilia reticulata*) (Evans et al. 2015) and bank voles (*Myodes glareolus*) (Mills et al. 2014), although the mechanisms behind these interactions are predominantly a matter of speculation.

Gryllid crickets have been an important model system for studying sexual selection (Alexander 1961; Bailey and Zuk 2008; Bretman et al. 2006; Hedrick and Dill 1993; Hunt et al. 2005; Rodríguez-Muñoz et al. 2008; Simmons 1986; Simmons 1992; Tregenza and Wedell 2002). In some cricket species, including the field cricket *Gryllus campestris,* both sexes dig burrows which serve as a refuge from predators (Rodríguez-Muñoz et al. 2011) and probably also from excessively hot or cold temperatures. We have previously demonstrated that both a male’s identity and the identity of his burrow contribute to the males’ likelihood of being paired with a female (Rodríguez-Muñoz et al. 2019d). Indeed, our earlier analysis revealed that burrow identity had a substantially larger influence than did the male’s own identity. A subsequent study of a German population of the same species (Niemelä et al. 2021) also found that both burrow and individual identity contribute to the attractiveness of males. However, while in our analysis, burrow was more important than individual ID, in their study, Niemelä et al. found similar proportions of variance explained by these two variables. Our previous analysis did not allow us to examine whether the greater importance of the burrow in our population was consistent across years, and Niemelä et al.’s (2021) study was of a single breeding season.

Our aims are to use 10 years of observations of the pairing behavior of a natural population of field crickets (*G. campestris)* (Rodríguez-Muñoz et al. 2010), to determine the relative contributions (to his pairing success) of male’s phenotype and of the burrows he occupies. This is possible because both sexes switch between burrows at intervals of a few hours to a few days, allowing us to estimate pairing success of males at a number of different burrows. First, we estimated the relative contribution of a male and the burrow he occupied on a male’s pairing success (i.e., hourly probability to share a burrow with a female). Second, we tested whether estimates of the relative amount of variation in male pairing success explained by the male and the burrow varied across years. Finally, we estimated effects of the interaction between a male and the burrow he occupied.

## METHODS

We have monitored the population of *G. campestris* in our meadow in northern Spain (see *WildCrickets.org*) since 2006 (Makai et al. 2020; Rodríguez-Muñoz et al. 2019b; Rodríguez-Muñoz et al. 2019c; Rodríguez-Muñoz et al. 2010). These field crickets have a single annual generation; nymphs of both sexes dig burrows in the autumn and overwinter in them, emerging to resume foraging and growth in early spring (Rodríguez-Muñoz et al. 2011). The first adults appear in our meadow in late April, and a few days after becoming adult, males begin to call by rubbing their forewings together. This calling attracts females (Zuk and Simmons 1997), but males also move around the meadow (Rodríguez-Muñoz et al. 2019d) encountering conspecifics at burrows where adults of both sexes spend the vast majority of their time. Both sexes compete for burrows: when two members of the same sex meet at a burrow, either one of them immediately leaves, or there is a fight, which is followed by the loser leaving. When members of the opposite sex meet at a burrow, fights are very rare; normally either one of them leaves, or the pair begin to cohabit at the burrow, frequently mating repeatedly during this period (Rodríguez-Muñoz et al. 2011). After an average of 0.64 ± 1.44 days (Rodríguez-Muñoz et al. 2019d), one of the pair moves to a different burrow. Males show no signs of attempting to prevent females from leaving, suggesting that females remaining at a burrow with a male are choosing to do so. Both sexes typically have multiple mating partners throughout their lives (Rodríguez-Muñoz et al. 2010). Burrows that are not occupied by a cricket are rapidly taken over by other invertebrates such as spiders and ants; typically an unoccupied burrow will be unrecognizable as a burrow within a few days and re-occupation of such sites by a cricket is very unusual. Similarly, burrows do not persist from one breeding season to the next.

We record information about the behavior of adult crickets by searching for burrows at least weekly from February each year until the end of the breeding season, when the last adult cricket dies, sometime in July. Each burrow is flagged with a unique number. A few days after emerging as an adult, we trap every individual at their burrow and glue a plastic tag onto their pronotum. The tag has a unique 1-2 character code allowing them to be visually identified. By mid to late April, usually before the adults start to emerge, we install up to 133 infrared day/night cameras. The cameras use motion activated digital video recording software (i-Catcher, i-codesystems.co.uk), to continuously record the activity around the entrance to each burrow and store these video recordings on servers housed in a building adjacent to the meadow. Because the number of occupied burrows is sometimes greater than the number of cameras, we carry out direct daytime observations of burrows that lack a camera every 1–2 days. We record the ID of any adult present or whether a nymph is in residence. This allows us to accurately record adult emergence dates, even if burrows are not directly monitored by video at that particular time (last instar nymphs and recently emerged adults rarely move among burrows, so the presence of an adult where there was a nymph the day before indicates an emergence). Our analysis covers the years 2006–2016 with the exception of 2014 (we had not completed detailed video watching for 2014 at the point when the analysis for this study was carried out).

### Ethical note

The crickets used in this study are removed from the meadow for a period of a maximum of a few hours during which time we take a small hemolymph sample, remove the tip of a hind leg, and attach a plastic tag by gluing it to the pronotum. Observations of individuals immediately after these procedures indicate that they exhibit normal behaviors within a few minutes of being released, and a study using heavier PIT tags glued to the same species of wild cricket (Niemelä et al. 2021) found no effect on survival. Our tagged crickets live out their natural lives in the meadow.

### Statistical analysis

We generated a binary hourly pairing success variable by defining, for each hour, whether a focal male shared a burrow with a female within that hour (1 = yes, 0 = no) (Niemelä et al. 2021). The final sample size was 201055 (hourly) data points for 511 males, occupying 1183 unique burrows, over a 10-year period (2006–2013 and 2015–2016).

We used mixed effects models to estimate the relative contribution to a male’s pairing success of male identity and the identity of the burrow he occupied. This allowed us to ask whether male phenotype or characteristics of the burrow he occupies explain more variation in pairing success. All models included hourly pairing success as a binary response variable, and male identity and burrow identity as random effects. To control for potential age effects, we included age (number of days since emerging as an adult) and squared age as covariates (both age and age^2^ can have independent effects) (Rodríguez-Muñoz et al. 2019b; Verburgt et al. 2011). We also fitted maximum age (adult lifespan) and squared maximum age in the models as a covariate, to control for selective disappearance of individuals (Bouwhuis et al. 2009; Niemelä et al. 2021). To answer our main question, i.e., whether the relative importance of the male himself versus his burrow in pairing success varied across years, we estimated year-specific individual and burrow variances. This was done by fitting year as a fixed effect and estimating separate intercepts for each year for individual and burrow variances, i.e., heterogeneous individual and burrow variances according to year. To control the parameter estimates for temporal variation, we included date (734 days) and hour (to control for within-day temporal variation; 24 levels) as random effects.

We were also interested in how much each unique male-burrow-combination explains variation in pairing success. To estimate this, we used an identical model structure as for the first model described above with one additional component; we generated a unique identity for each male-burrow combination and fitted it as an additional random effect in the model (3869 levels, i.e., unique burrow-male combinations). We present results from both the simpler model without this burrow-male combination effect and the more complex model because it is biologically interesting to examine the relative contributions of male and burrow even if the combination of these factors can also have an effect.

Since hourly pairing success is a binary variable, we used a logistic Bernoulli link function which means that the residual variance was fixed to π^2^/3 (Nakagawa and Schielzeth 2010). The proportion of variance in pairing success explained by each focal random effect, i.e., the repeatability for each random effect, was calculated as the focal random effect variance divided by the total phenotypic variance (Falconer and Mackay 1996). Since 95% credible intervals often fail to find true significant differences between two distributions when a 0.05 *P*-value threshold is being used (Goldstein and Healy 1995; Schenker and Gentleman 2001), we used the often suggested 83% credible intervals to test whether two repeatabilities differ from each other (Austin and Hux 2002; MacGregor-Fors and Payton 2013; Payton et al. 2003). If the 83% credible intervals did not overlap between the two focal estimates, we concluded that the difference between the two was statistically significant (Austin and Hux 2002; MacGregor-Fors and Payton 2013; Payton et al. 2003). Additionally, we tested whether two focal repeatabilities differ significantly by calculating their ∆-posterior distribution, where one focal posterior distribution for male repeatability (e.g., for male identity repeatability for year 2010) was subtracted from the posterior distribution for burrow repeatability (e.g., burrow identity repeatability for year 2010). ∆-estimates with 95% Cl’s not overlapping zero were interpreted as statistically significant (Royauté et al. 2015; Royauté and Dochtermann 2021).

All models were run using Bayesian mixed effects models with the brms package (version 2.14.4) (Bürkner 2017), using a binomial (Bernoulli; logit) link function. We used default Student-T priors and the models were run with 2500 iterations with 400 burn-in and sampling rate of 2. All models were run in the R statistical environment (version 3.6.3) (R Development Core Team 2020). According to posterior predictive checks, both models performed well (Supplementary Tables S2 and S3).

## RESULTS

### Descriptive statistics

**Figure 1:**
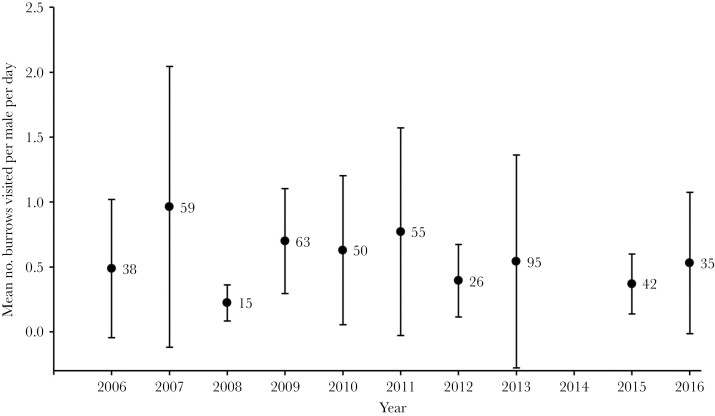
Grand mean of the mean number of different burrows each male occupied for each year (males live only one year so years are completely independent). Error bars are standard deviations; the number next to each point is the total number of males in the sample from that year. We only included males that were monitored for a total time of at least 24 h during their adult life.

**Figure 2:**
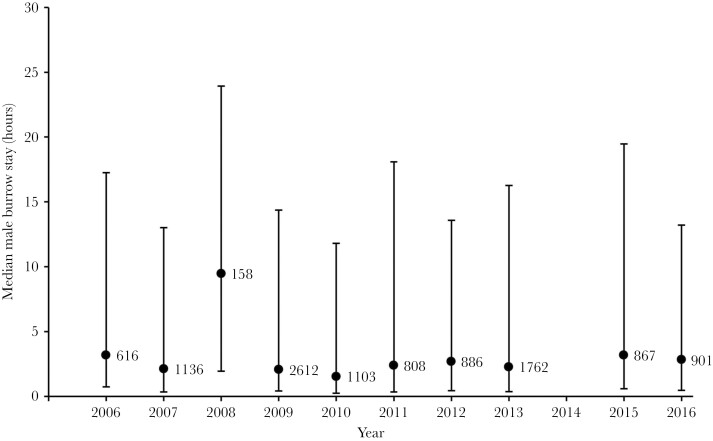
The median duration that males spent on each visit to a burrow in each year. These are unique visits, so they include repeat visits to the same burrow. Error bars show the quartiles; the number next to each point is the total number of unique visits to burrows made by all males combined in that year.

**Figure 3:**
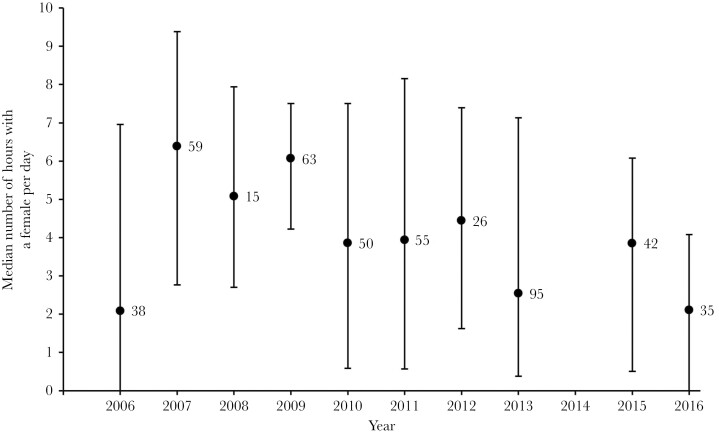
The median number of hours that males spent sharing a burrow with a female per day each year. Females are considered resident at the burrow for the focal hour providing they have occupied the focal burrow at any time within that hour. Error bars show the quartiles; the number next to each point is the total number of males in the sample from that year. We only included males that were monitored for at least 24 h during their adult life.

Males moved around the meadow occupying multiple burrows (Figure 1). There was variation among years in how many times males moved among burrows, with some years in which males had five times as many periods of residency at different burrows as other years (Figure 1). There was also variation among years in the average length of time males spent at a burrow before moving to a different one (Figure 2). As one might expect, the year with the smallest number of burrow visits was also the year with the longest mean duration of stay each burrow. However, it is clear from glancing at Figures 1 and 2 that there is also independent variation in these two parameters; for instance, 2016 has the second highest median number of burrows visited but does not have a below average mean stay duration. There is also among year variation in how much time males spend with a female (Figure 3.).

### Variation in male pairing success explained by male versus burrow

When comparing the proportion of variance explained by male identity and burrow identity to each other across years using 83% credible intervals, burrow identity explained proportionally more variation in male pairing success than male identity in 8 out of 10 years (Table 1). Moreover, in the 2 years when the difference was not statistically significant, the significance was close to the threshold and the point estimates for burrow repeatability were always higher. This means that a male’s pairing success is always more dependent upon the properties of the burrow, than on the properties of the male himself. This result was confirmed by using ∆-posterior distributions for the comparisons (last column of Table 1). All the estimated standard deviations for all random effects can be found in Table 2.

**Table 1:** The proportion of variance explained (repeatability, R) for burrows and individuals for each year and 83% credible intervals (CI); whether the greater repeatability of burrow vs individual is significant (Yes, No); Differences between burrows and individuals (∆R) and 95% credible intervals (CI)). Repeatabilities were calculated from model estimates presented in Table 2

Year	Burrow	Individual	R_burrow_ > R_ind._	∆R: R_burrow_-R_male_(95% CI)
	R (83% CI)	R (83% CI)		
2006	0.37 (0.30, 0.46)	0.23 (0.16, 0.33)	No	0.15 (-0.09, 0.35)
2007	0.41 (0.36, 0.48)	0.15 (0.11, 0.20)	Yes	0.27 (0.12, 0.39)
2008	0.48 (0.35, 0.62)	0.29 (0.17, 0.44)	No	0.19 (-0.21, 0.52)
2009	0.30 (0.25, 0.35)	0.08 (0.06, 0.11)	Yes	0.22 (0.13, 0.31)
2010	0.39 (0.33, 0.46)	0.19 (0.14, 0.26)	Yes	0.20 (0.03, 0.35)
2011	0.52 (0.45, 0.59)	0.21 (0.16, 0.28)	Yes	0.30 (0.10, 0.47)
2012	0.44 (0.35, 0.54)	0.15 (0.09, 0.25)	Yes	0.29 (0.05, 0.50)
2013	0.42 (0.37, 0.48)	0.28 (0.23, 0.34)	Yes	0.14 (>-0.01, 0.28)
2015	0.47 (0.39, 0.56)	0.20 (0.14, 0.27)	Yes	0.27 (0.06, 0.45)
2016	0.48 (0.40, 0.56)	0.09 (0.05, 0.14)	Yes	0.39 (0.23, 0.54)

**Table 2:** Sources of variation in hourly male mating success: we present posterior modes for fixed (β) and random (σ) parameters 95% credible intervals, derived from univariate mixed effects model (note that in binary (logistic) models residual variance is fixed to π^2^/3 (see Methods)

Fixed effects*	β (95% CI)	Random	Effects	σ (95% CI)
Age	0.094 (0.081, 0.107)	Date	2.162 (2.009, 2.329)	
Age2	>−0.001 (−0.001, −0.001)	Hour	0.313 (0.235, 0.432)	
Maximum age	<0.001 (−0.0364, 0.0399)			
Maximum age2	<0.001 (−0.001, 0.001)	Year	Individual	Burrow
Intercept	−5.885 (−7.245, −4.604)	2006	2.207 (1.572, 0.049)	2.805 (2.242, 3.547)
Year_2007_	0.724 (−0.767, 2.268)	2007	1.710 (1.347, 2.169)	2.813 (2.392, 3.289)
Year_2008_	0.590 (−2.270, 3.253)	2008	3.341 (2.099, 5.141)	4.265 (3.047, 5.998)
Year_2009_	1.425 (−0.017, 2.903)	2009	1.033 (0.835, 1.281)	1.993 (1.662, 2.320)
Year_2010_	−0.553 (−2.155, 1.050)	2010	1.964 (1.503, 2.562)	2.786 (2.313, 3.341)
Year_2011_	−0.191 (−1.966, 1.509)	2011	2.614 (2.061, 3.351)	4.049 (3.371, 4.886)
Year_2012_	−0.075 (−1.860, 1.797)	2012	1.792 (1.156, 2.710)	3.030 (2.282, 3.980)
Year_2013_	1.103 (−0.461, 2.816)	2013	2.778 (2.304, 3.333)	3.411 (2.940, 3.956)
Year_2015_	−0.416 (−2.136, 1.394)	2015	2.252 (1.739, 2.963)	3.449 (2.777, 4.273)
Year_2016_	−0.473 (−2.132, 1.131)	2016	1.307 (0.914, 1.824)	3.030 (2.422, 3.813)

*Year 2006 is the reference year.

When we added the unique combination of burrow and male as an additional random effect to the model described in Table 2, it explained a higher proportion of variation in male pairing success compared to male across years when 83% credible intervals were compared (Table 3). This means that a combination of male phenotype and the environment he occupies explains more variation in male pairing success than male phenotype or burrow alone. The point estimates of the proportion of variance explained (repeatability) were also always larger for the combination of male phenotype and the environment he occupies than the environment alone (Table 3). However, the 83% credible intervals overlapped in some of the years (Table 3). These results were confirmed by using ∆-posterior distributions for the comparisons (Table 4). Estimated standard deviations for all random effects can be found in Table 5.

**Table 3:** The proportion of variance explained (repeatability, R) for burrows, individuals and unique burrow-year combination for each year and 83% credible intervals (CI). Repeatabilities calculated from model estimates presented in Table 5

Year	Burrow	Individual	Burrow-individual
	R (83% CI)	R (83% CI)	R (83% CI)
2006	0.082 (0.011, 0.186)	0.056 (0.001, 0.135)	0.532 (0.415, 0.645)
2007	0.270 (0.201, 0.353)	0.059 (<0.001, 0.030)	0.353 (0.295, 0.413)
2008	0.102 (0.005, 0.298)	0.100 (0.008, 0.254)	0.489 (0.303, 0.663)
2009	0.089 (0.053, 0.133)	0.004 (<0.001, 0.016)	0.521 (0.477, 0.565)
2010	0.075 (0.007, 0.165)	0.008 (<0.001, 0.042)	0.471 (0.379, 0.555)
2011	0.344 (0.246, 0.456)	0.009 (<0.001, 0.043)	0.378 (0.293, 0.472)
2012	0.126 (0.011, 0.272)	0.020 (0.001, 0.099)	0.415 (0.299, 0.556)
2013	0.182 (0.118, 0.258)	0.034 (0.003, 0.084)	0.450 (0.377, 0.519)
2015	0.247 (0.156, 0.348)	0.077 (0.022, 0.152)	0.363 (0.289, 0.444)
2016	0.210 (0.094, 0.322)	0.008 (<0.001, 0.045)	0.471 (0.367, 0.581)

**Table 4:** The difference in the proportion of variance explained by the burrow and the individual male (∆-repeatability, ∆R) and 95% credible intervals (CI)). ∆-Repeatabilities were calculated from model estimates presented in Table 5

Year	∆R: R_burrow_-R_individual_(95% CI)	∆R: R_burrow&individual_-R_individual_ (95% CI)	∆R: R_burrow&individual_-R_burrow_ (95% CI)
2006	0.03 (−0.12, 0.17)	0.48 (0.24, 0.66)	0.45 (0.16, 0.67)
2007	0.26 (0.15, 0.38)	0.34 (0.25, 0.43)	0.08 (−0.10, 0.24)
2008	<0.01 (−0.32, 0.36)	0.38 (−0.06, 0.70)	0.38 (−0.17, 0.71)
2009	0.08 (0.03, 0.15)	0.52 (0.45, 0.58)	0.43 (0.32, 0.53)
2010	0.06 (−0.03, 0.20)	0.46 (0.31, 0.58)	0.40 (0.14, 0.57)
2011	0.33 (0.18, 0.48)	0.36 (0.23, 0.49)	0.04 (−0.23, 0.28)
2012	0.09 (−0.09, 0.32)	0.38 (0.17, 0.58)	0.29 (−0.05, 0.58)
2013	0.14 (0.02, 0.26)	0.41 (0.26, 0.53)	0.27 (0.08, 0.44)
2015	0.17 (−0.03, 0.36)	0.28 (0.11, 0.44)	0.12 (−0.13, 0.33)
2016	0.19 (0.03, 0.36)	0.46 (0.29, 0.62)	0.26 (−0.03, 0.58)

**Table 5:** Sources of variation in hourly male mating success; we present posterior modes for fixed (β) and random (σ) parameters 95% Credible Intervals, derived from univariate mixed effects model where unique combination of a burrow and male was included as an additional random effect

Fixed effects*	β (95% CI)	Random	Effects	σ (95% CI)	
Age	0.151 (0.134, 0.168)	Date	1.799 (1.661, 1.934)		
Age2	−0.003 (−0.003, −0.002)	Hour	0.353 (0.263, 0.493)		
Maximum age	−0.033 (−0.067, 0.003)				
Maximum age2	0.001 (<0.001, 0.001)	Year	Individual	Burrow	Burrow-Individual
Intercept	−5.945 (−7.165, −4.756)	2006	1.097 (0.169, 2.076)	1.304 (0.140, 2.385)	3.388 (2.638, 4.225)
Year2007	1.216 (−0.069, 2.479)	2007	0.362 (0.014, 0.913)	2.240 (1.682, 2.826)	2.545 (2.215, 2.928)
Year2008	1.295 (−0.687, 3.359)	2008	1.601 (0.072, 3.391)	1.612 (0.112, 3.573)	3.509 (2.410, 4.953)
Year2009	1.030 (−0.204, 2.314)	2009	0.271 (0.012, 0.684)	1.249 (0.830, 1.690)	3.023 (2.764, 3.306)
Year2010	0.451 (−0.803, 1.665)	2010	0.406 (0.013, 1.019)	1.023 (0.137, 1.880)	2.680 (2.233, 3.185)
Year2011	0.200 (−1.276, 1.596)	2011	0.539 (0.025, 1.346)	2.987 (2.202, 3.863)	3.126 (2.597, 3.764)
Year2012	0.736 (−0.864, 2.142)	2012	0.632 (0.039, 1.651)	1.363 (0.100, 2.522)	2.605 (1.958, 3.359)
Year2013	0.662 (−0.600, 1.984)	2013	0.805 (0.093, 1.542)	1.907 (1.303, 2.545)	3.008 (2.633, 3.416)
Year2015	−0.309 (−1.701, 1.177)	2015	1.296 (0.356, 2.137)	2.338 (1.622, 3.241)	2.832 (2.365, 3.375)
Year2016	−0.326 (−1.770, 1.176)	2016	0.482 (0.018, 1.323)	2.116 (0.959, 3.091)	3.227 (2.607, 3.974)

*Year 2006 is the reference year.

Our analysis shows male age and squared age make significant contributions to male pairing success (Tables 2 and 5), which increases with age with a slightly convex shape (Supplementary Figure S1). The effects of maximum age were nonsignificant indicating a lack of age-selective disappearance of individuals from the data (Tables 2 and 5).

## DISCUSSION

Male reproductive success is dependent on mating with females, which in *G. campestris* requires the male to spend time with a female at a burrow (Rodríguez-Muñoz et al. 2010). Our previous work demonstrated that cohabiting with a female is significantly positively associated with number of offspring in future generations (Rodríguez-Muñoz et al. 2019d) even though it is not unusual for a pair to share a burrow without a mating occurring. Also, we previously identified a negative relationship between the total length of each period of cohabitation and the mating rate of the cohabiting pair, so our analysis does not capture the full complexity of sexual selection in the system. Nevertheless our study confirms the findings of our previous analysis (Rodríguez-Muñoz et al. 2019d), and that of an independent study of a German population of the same species (Niemelä et al. 2021); that the burrow a male occupies is a better predictor of whether the male will be paired with a female, than is his own identity.

Because male crickets have such striking calling and courtship songs, it is easy to assume that these secondary sexual traits are dominant variables in relation to sexual selection in this species. However, our results (Figures 1 and 2.) show that rather than simply remaining at a burrow and singing to attract females, males move frequently between burrows, engaging in potentially costly fights when they encounter rival males (Fisher et al. 2016, 2019; Rodríguez-Muñoz et al. 2010), and presumably exposing themselves to increased predation risk while moving between burrows. Our analysis provides insights into the surprisingly dynamic relationship between crickets and burrows: The combination of individual male identity and burrow identity was an even better predictor of pairing success than either of these factors alone.

Before attempting to understand why it is the unique combination of male and burrow that best predicts pairing success, we first need to understand what makes a particular burrow more attractive, and how this might interact with the identity of a male. One possibility is that burrows themselves can be regarded as a secondary sexual trait; males have to fight to gain and retain control of them, and hence occupancy of sought-after burrows may provide females with information about the genetic quality of the male. Hence, it may be arbitrary features of burrows that are preferred. Alternatively, there may be more predictable features of burrows that make some of them more attractive. In some species, such as Wellington tree wetas (*Hemideina crassidens*), tree galleries provide food resource to females, and larger males reside in galleries housing larger groups of females (Kelly 2005). However, in *G. campestris*, burrows are solely a refuge from predation and weather, and do not have any obvious intrinsic features that might affect their value. Also, features of the burrow do not appear likely to affect how easy they are to take-over or defend—fights for burrows occur outside the burrow. Burrow location is a potential indicator of quality, both because it may be valuable to be close to conspecifics, and because high burrow density might be expected in better locations. The position of the burrow did not have any measured effect on attractiveness in a lek-forming mole cricket species (Howard et al. 2011). However, in other insects, the position of territories is directly related to their attractiveness. For instance, in the medfly (*Ceratitis capitata*), males in the highest positions in the lek were more likely to gain matings (Niyazi et al. 2008), and in the mosquito (*Anopheles gambiae*), the spatial location of swarms affected their attractiveness to males and females (Diabaté et al. 2011). More attractive burrows may then attract the attention of more individuals and end up being held by higher quality males. In collared flycatchers, males with preferred visual signaling phenotypes were more successful in acquiring high-quality territories (Pärt and Qvarnström 1997).

There are numerous reports of correlations between phenotypic traits considered to be indicative of male quality and measures of territory quality in passerine birds (See references in Lambrechts and Dhondt 1988) (although such findings are not universal and it has been argued that they are less common in non-migratory species [Lambrechts and Dhondt 1988]). T It is possible that a similar situation occurs in field crickets; if more attractive males, are more likely to spend time at more attractive burrows, the combined burrow-male random effect would be expected to explain more variance than either male or burrow identity alone. However, it is also possible that there is an interaction between male identity and burrow identity affecting how much time females spend there. We find ourselves in a similar position to the majority of studies that have identified genotype by environment interactions affecting the expression of sexual traits species (e.g., Evans et al. 2015; Hunt and Hosken 2014; Mills et al. 2014; Plesnar-Bielak et al. 2018); generally the mechanisms behind these interactions are predominantly a matter of speculation.

One of the striking observations from our 10 + years of studying this natural population, is just how much environmental and demographic variation there is within and among years. We are only able to record some of the major consequences of this variation for our population, but even these simple parameters reveal striking differences among years. Examples that we have reported previously include: differences in sex ratio among the years, which range from years with an equal number of each sex to years with twice as many females as males (Rodríguez-Muñoz et al. 2019a); and differences in the average lifespan of males, which in the data analyzed here, range from 30.4 days (SD = 17.9) in 2009 to just under 20 days in 2006, 2011 and 2013. It is tempting to speculate about how these demographic parameters might cause the among-year variation in mating system dynamics seen in Figures 1–3. Similarly, there are a numerous environmental variables (e.g., over-wintering temperatures, predator and parasite dynamics, rainfall during the breeding season, etc.) that might affect these dynamics. However, there are such a large number of potential causative relationships between an almost unlimited number of variables that even 10 years of data are unlikely to be enough to reach any reliable conclusions, especially given the extensive within-year variation that is evident from the error bars in Figures 1–3.

We have previously shown that older males are more likely to be paired with females (Rodríguez-Muñoz et al. 2019d) and similar findings have been reported for a population of crickets in southern Germany (Niemelä et al. 2021). These findings are supported by the present analyses with age and squared age making significant contributions to male pairing success (Tables 2 and 6).

The insights our analysis provides, are firstly, that even in animals with striking secondary sexual traits, more cryptic and environmentally dependent factors are likely to play a major role in sexual selection. In our crickets, the importance of burrow identity may explain why males are willing to expose themselves to the risk of moving among burrows. A high-quality male may fail to reach his potential if he stays at the burrow in which he overwintered, so it may pay to move among burrows looking for one that is more likely to attract a female. Secondly, the discovery of strong effects on pairing success, of the combination of male identity and burrow identity suggest that interactions between sexual selection and the environment in which it occurs may be complex and easy to overlook.

## Supplementary Material

arac067_suppl_Supplementary_Material
