# Supplementary material for: Environment and mate attractiveness in a wild insect
Source: Behav Ecol. 2022 Jul 26;33(5):999–1006. doi: 10.1093/beheco/arac067 (PMC9639583; doi:10.1093/beheco/arac067)
Supplement: arac067_suppl_Supplementary_Material [file arac067_suppl_supplementary_material.docx]

# Supplementary Information


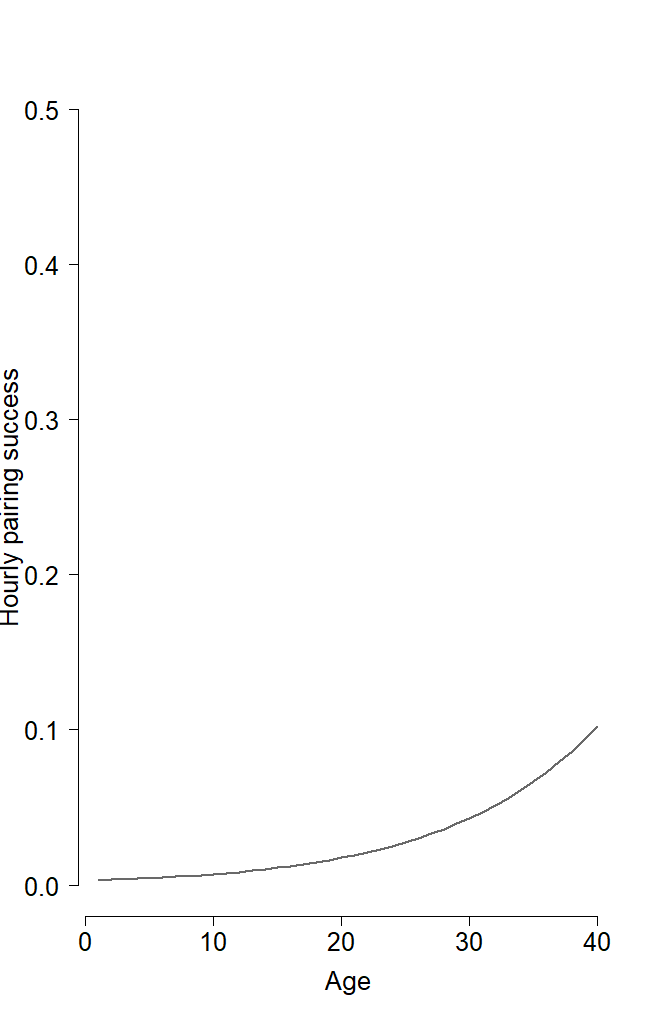


**Supplementary Figure S1**. Model prediction for population level regression line (black line) representing how hourly pairing success changes as a function of age. Estimates, i.e. linear and non-linear age effects, for the regression line are taken from the model presented in the last column of Table 1.


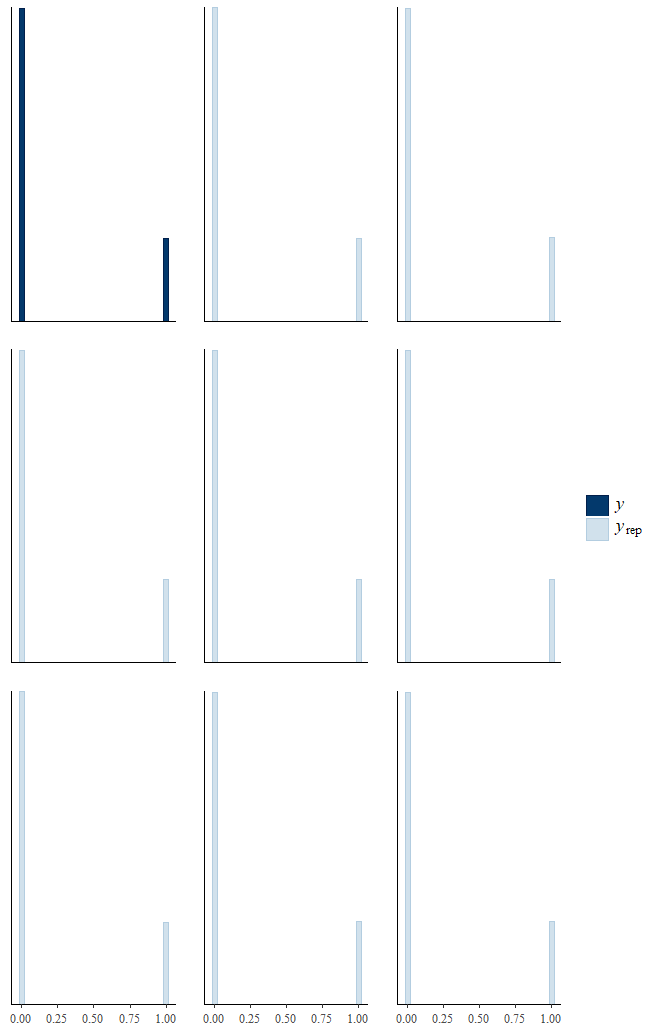


**Supplementary Figure S2**. Results of posterior predictive check for the model with male and burrow identities as random effects. Dark blue columns (1 panel) represent the distribution of the actual, binary, data and the light blue columns (8 panels) represent the distributions of the data that is simulated based on the model estimates. Since distributions of both the actual and simulated data looks identical, we conclude that the model performed well.


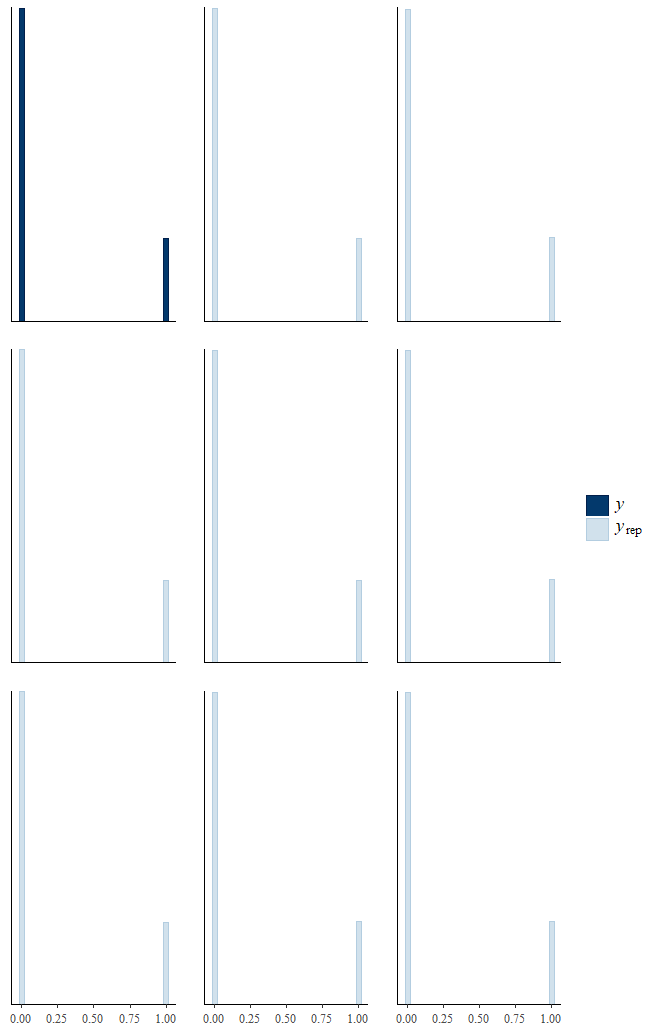


**Supplementary Figure S3.** Results of posterior predictive check for the model with male and burrow identities, as well as unique male-burrow combination, as random effects. Dark blue columns (1 panel) represent the distribution of the actual, binary, data and the light blue columns (8 panels) represent the distributions of the data that is simulated based on the model estimates. Since distributions of both the actual and simulated data looks identical, we conclude that the model performed well.
